# Optimizing hybrid vigor: a comprehensive analysis of genetic distance and heterosis in eggplant landraces

**DOI:** 10.3389/fpls.2023.1238870

**Published:** 2023-08-31

**Authors:** Neha Rajan, Sandip Debnath, Kahkashan Perveen, Faheema Khan, Brijesh Pandey, Akanksha Srivastava, Mehrun Nisha Khanam, Vetriselvan Subramaniyan, Vinoth Kumarasamy, Pronob J. Paul, Mohan Lal

**Affiliations:** ^1^ Department of Genetics and Plant Breeding, Institute of Agriculture, Visva-Bharati University, West Bengal Sriniketan, India; ^2^ Department of Botany & Microbiology, College of Science, King Saud University, Riyadh, Saudi Arabia; ^3^ Krishi Vigyan Kendra, Banda University of Agriculture and Technology, Mahoba, India; ^4^ Department of Biotechnology, ICAR-Indian Institute of Rice Research, Hyderabad, India; ^5^ School of Biological Sciences, Seoul National University, Seoul, Republic of Korea; ^6^ Pharmacology Unit, Jeffrey Cheah School of Medicine and Health Sciences, Monash University, Malaysia, Bandar Sunway, Selangor Darul Ehsan, Malaysia; ^7^ Center for Transdisciplinary Research, Department of Pharmacology, Saveetha Dental College, Saveetha Institute of Medical and Technical Sciences, Saveetha University, Chennai, Tamil Nadu, India; ^8^ Department of Parasitology and Medical Entomology, Faculty of Medicine, Universiti Kebangsaan Malaysia, Kuala Lumpur, Malaysia; ^9^ International Rice Research Institute, South Asia Hub, Hyderabad, India; ^10^ Agrotechnology and Rural Development Division, CSIR-NORTH-EAST INSTITUTE OF SCIENCE AND TECHNOLOGY, Jorhat, Assam, India

**Keywords:** genetic diversity, heterosis, ssr markers, genetic distance, hybrid vigor, egg plant

## Abstract

**Introduction:**

This study explored the molecular characterization of 14 eggplant (brinjal) genotypes to evaluate their genetic diversity and the impact of heterosis. As eggplant is a vital horticultural crop with substantial economic and nutritional value, a comprehensive understanding of its genetic makeup and heterosis effects is essential for effective breeding strategies. Our aim was not only to dissect the genetic diversity among these genotypes but also to determine how genetic distance impacts heterotic patterns, which could ultimately help improve hybrid breeding programs.

**Methods:**

Genetic diversity was assessed using 20 SSR markers, and the parental lines were grouped into five clusters based on the Unweighted Pair Group Method of Arithmetic Means (UPGMA). Heterosis was examined through yield and yield-related traits among parents and hybrids.

**Results:**

Polymorphisms were detected in eight out of the twenty SSR markers across the parental lines. Notably, a high genetic distance was observed between some parents. The analysis of yield and yield-related traits demonstrated significant heterosis over mid, superior, and standard parents, particularly in fruit yield per plant. Two crosses (RKML-26 X PPC and RKML1 X PPC) displayed substantial heterosis over mid and better parents, respectively. However, the positive correlation between genetic distance and heterosis was only up to a certain threshold; moderate genetic distance often resulted in higher heterosis compared to very high genetic distance.

**Discussion:**

These findings emphasize the critical role of parental selection in hybrid breeding programs. The results contribute to the understanding of the relationship between genetic distance and heterosis, and it is suggested that future research should delve into the genetic mechanisms that drive heterosis and the effect of genetic distance variance on heterosis. The insights drawn from this study can be harnessed to enhance crop yield and economic value in breeding programs.

## Introduction

The recent progress in genetic technologies has provided unparalleled prospects to enhance the genetic variability of eggplant. Marker-assisted selection (MAS) techniques have been developed to facilitate the identification, selection, and crossing of plants with desirable traits by breeders. These techniques have been found to be highly efficient in achieving this goal. The utilisation of these techniques may facilitate an increased comprehension of the eggplant’s genetic composition and expedite the breeding procedure to augment the crop’s efficacy and durability (Nunome et al., 2009; Wang et al., 2010; Adeniji et al., 2012). The utilisation of SSR markers has proven to be an indispensable technique in the field of diversity studies. This is attributed to their remarkable polymorphism, co-dominance, and facile detection through Polymerase Chain Reaction (PCR) methods (Gantait et al., 2014; Debnath and Guha, 2015; Chourey et al., 2017).

Recent technological advancements have facilitated a more in-depth exploration of the mechanisms that underlie heterosis, also known as hybrid vigor. This phenomenon is characterised by the superior traits exhibited by the first generation of hybrid offspring, surpassing those of the most proficient parent (Schnable and Springer, 2013; Huang et al., 2015; Wallace et al., 2018; Tomkowiak et al., 2020; Monforte, 2021; Chakraborty et al., 2022). The phenomenon of heterosis, which is widely acknowledged to be intricate, is thought to be impacted by modifications in the structure of the genome, including the prevalence and distribution of particular gene families (Song and Messing, 2003; Huang et al., 2015). Nonetheless, the implications for the amelioration of crops are significant. The phenomenon of hybridization in plants has been observed to result in enhanced growth, yield, and resilience, surpassing those of the parental plants. This characteristic of hybrid plants could be exploited to augment the productivity and nutritional quality of aubergine.

The diversification of eggplant’s genetic composition is not solely a scientific pursuit, but also a crucial economic necessity. Eggplant, a vegetable with origins traced back to India, is a significant contributor to agricultural land usage and yield in the country. According to Sharma and Kaushik (2021), eggplant accounts for 8.14% of agricultural land usage and contributes to 9% of the total yield. Notwithstanding the cultural and nutritional importance of the plant, aubergine cultivation is not bereft of obstacles. Numerous aubergine cultivars exhibit susceptibility to various pests and diseases, while their nutritional composition displays considerable variability. Augmenting genetic diversity can potentially mitigate these concerns by endowing the crop with the capacity to endure adversities and furnish reliable, superior nourishment (Satpathy et al., 2021).

Therefore, the presence of genetic diversity is an indispensable resource in our efforts to address the issue of food insecurity and enhance nutritional achievements. The utilisation of this technique enables the augmentation of the robustness, yield, and nourishing properties of significant cultivars such as *Solanum melongena*. The utilisation of genetic technologies and a comprehensive comprehension of mechanisms such as heterosis can enable the exploitation of eggplant’s complete genetic diversity to the advantage of farmers, consumers, and the environment. Given the concurrent challenges of population expansion and climate change, the significance of this research cannot be overemphasized.

The present investigation aims to elucidate the complex interplay between genetic similarity and hybrid vigor in *Solanum melongena* (brinjal or aubergine), with the ultimate goal of devising more accurate predictive models for heterosis effects in hybrid cultivars. The present study entails a comprehensive investigation of the copious genetic variability present in brinjal, specifically in India, which is one of its primary centres of origin. The objective is to harness this diversity for the amelioration of brinjal cultivars via heterosis breeding. The present investigation integrates the utilisation of molecular markers, particularly Simple Sequence Repeats (SSRs), to expedite a more efficient breeding methodology. The primary aim of this study is to utilise the obtained results to augment crop efficacy, amplify output, boost immunity against diseases, and thereby aid in worldwide efforts to tackle food scarcity by creating resilient, high-capacity eggplant varieties.

## Materials and methods

In this study, the methodology was designed around three primary areas of focus: plant materials, morphological characterization, molecular characterization, and heterosis estimation.

### Plant materials

The investigation took place at the RKMVERI’s Ranchi research farm over three Rabi seasons (2017-18, 2018-19, and 2019-20). A total of 35 indigenous brinjal lines sourced from various locations in Jharkhand were used, alongside four high-yielding genotypes, selected as testers. These lines were carefully chosen from a core collection pool for their distinctive characteristics and their adherence to the DUS (distinctness, uniformity, and stability) standards as prescribed by the Authority for the Protection of Plant Varieties and Farmers’ Rights, under the Indian Ministry of Agriculture and Farmers Welfare. Post-classification and further evaluation, these lines are slated for registration (Guidelines for the conduct of the test for DUS on Brinjal/Egg plant, 2009; Rajan et al., 2020).

### Morphological characterization

A Randomized Block Design was used to conduct the experiment thrice, considering 19 quantitative characteristics as outlined by DUS guidelines. Four-week-old seedlings were transplanted into the main field with a spacing of 75 X 50 cm. Each plot comprised 20 plants across a 9 m2 area, and five randomly chosen plants from each plot were assessed for all quantitative traits across all replications. Exceptions were the days to flower initiation, days to 50% flowering, and days to fruit initiation, which were monitored at the plot level. The mean of each trait was computed over the three replications, and a Euclidean dissimilarity matrix was generated using these traits. Accessions were then clustered using Ward’s method, facilitated by the R ‘cluster’ package.

### Molecular characterization

The genetic diversity of ten indigenous brinjal lines and four testers were evaluated using 20 SSR (Simple Sequence Repeats) markers (Nunome et al., 2003). DNA was isolated from fresh leaves using the CTAB extraction technique (Doyle, 1990). The PCR process involved an initial denaturation at 94°C for 4 minutes, 35 cycles of denaturation at 94°C for 30 seconds, annealing at 65°C for 30 seconds, extension at 72°C for 1 minute, and a final extension at 72°C for 7 minutes. The amplified fragments were separated and visualized using 2% agarose gel and Ethidium Bromide staining. Each genotype was scored based on the presence (1) or absence (0) of the indicated allele for each primer. A dendrogram was then generated using the unweighted pair group method with arithmetic average (UPGMA), and the genetic relationship between the 14 genotypes was assessed. Data analysis was carried out using the NTYSYS-pc program (Rohlf, 2000), and polymorphism information content (PIC) and gene diversity were estimated using Power Marker ver. 3.25 (Liu and Muse, 2005).

### Estimation of heterosis

The assessment of heterosis, also known as hybrid vigor, is a crucial aspect in augmenting agricultural productivity and acclimatising to alterations in the environment (East, 1936; Shull, 1952). The current investigation was designed to assess heterosis in brinjal crops using a well-documented mating strategy known as the Line X Tester design. This approach involves selecting specific parent lines (lines) and crossing them with chosen tester lines (testers). In a Line x Tester design, the ‘line’ is typically a group of inbred lines, and the ‘tester’ is a group of genetically distinct inbred lines. The lines are crossed with the testers, and the offspring from these crosses (hybrids) are evaluated for their performance. To facilitate this, we selected parental lines, both female (lines) and male (testers), based on their distinct traits and genetic variability. Each of the selected ‘lines’ was mated with each of the ‘testers’ in a systematic manner. The resulting crosses or hybrids were then grown and evaluated for their heterotic performance. (Sprague and Tatum, 1942; Kempthorne, 1969; Chand et al., 2022). Heterosis, or hybrid vigor, is calculated by comparing the hybrid’s performance to its parents’ performance. There are a few different ways heterosis can be calculated, depending on what aspect of parental performance are of interest. Here are three commonly used formulas for calculating heterosis:

#### Mid-parent heterosis

This is calculated by comparing the performance of the hybrid to the average performance of its parents.


$$\text{Formula}:\text{ Mid}-\text{parent heterosis }(\%)=[({\text{F}}_{1}-\text{MP})/\text{MP}]\times 100$$

Here, F_1_ represents the value of the trait in the hybrid, and MP represents the average value of the trait in the parents.

#### Better-parent heterosis (or best-parent heterosis)

This is calculated by comparing the performance of the hybrid to the performance of the better-performing parent.


$$\text{Formula}:\text{ Better}-\text{parent heterosis }(\%)=[({\text{F}}_{1}-\text{BP})/\text{BP}]\times 100$$

Here, F_1_ is the value of the trait in the hybrid, and BP is the value of the trait in the better-performing parent.

#### Standard Heterosis

This is calculated by comparing the performance of the hybrid to a standard cultivar or check variety.


$$\text{Formula}:\text{ Standard heterosis }(\%)=[({\text{F}}_{1}-\text{check})/\text{check}]\times 100$$

Here, F_1_ represents the value of the trait in the hybrid, and ‘check’ represents the value of the trait in the standard cultivar. These formulas allow the heterosis for a specific trait to be expressed as a percentage, which can be helpful in comparing the degree of heterosis across different hybrids or different traits.

Ten indigenous brinjal lines were selected from a pool of 35 lines sourced from different locations in Jharkhand, based on their cluster affiliations. The chosen lines were found to be representative of all four clusters, namely RKML1 for Cluster 1, RKML 2, 3, 6 & 26 for Cluster 2, RKML4, 7, 11 & 34 for Cluster 3, and RKML 5 for Cluster 4. This selection was made to ensure a diverse genetic foundation for the study. In accordance with the study conducted by earlier workers (Kamalakkannan et al., 2007), four unique and currently existing genotypes, recognised for their broad genetic base and with wider adaptability, were selected as testers. These genotypes include Pusa Purple Cluster, Pusa Purple Long, Swarna Pratibha, and Swarna Shyamli.

A series of crosses were performed between ten genetically distinct lines and four tester lines. In order to achieve a bountiful and thriving harvest, customary cultural procedures were executed in accordance with the guidelines established by Birsa Agricultural University (BAU), a well-established methodology for enhancing agricultural output (Birsa Agricultural University, Kanke, Ranchi, Jharkhand and Central Research Institute for Dry Land Agriculture (CRIDA), HYDERABAD, 2019).

The present investigation concerns a heterosis study, wherein multiple quantitative traits were assessed on a sample of five arbitrarily chosen plants in every treatment. The study encompassed an analysis of various traits, namely the number of primary branches, leaf length, leaf width, petiole length, fruit length, fruit diameter, number of fruits per plant, number of marketable fruits per plant, single fruit weight, seeds per fruit, yield per plant, marketable yield per plant, and total yield per hectare. The assessment of traits is a crucial aspect in the identification of exceptional hybrids within plant breeding initiatives (Falconer and Mackay, 1996).

Phenological traits, including the duration of time until the initiation of flowering, the duration of time until 50% of flowering, and the duration of time until the initiation of fruiting, were observed and documented on a complete plot level. The prompt and accurate observation of these traits is of utmost importance as they have a direct impact on the crop’s productivity and adaptability to particular cultivation conditions (Craufurd and Wheeler, 2009).

The present study aimed to estimate heterosis and assess the potential of selected brinjal lines crossed with specific testers for further use in breeding programmes, by analysing hybrid vigor. The aforementioned endeavours were aimed at providing insights into methodologies for enhancing the quality of brinjal crops, thereby aiding in the resolution of the worldwide issue of insufficient food supply, as emphasised by the International Treaty on Plant Genetic Resources for Food and Agriculture (Moore and Tymowski, 2005).

## Results

### Divergence in parental lines

**Figure 1:**
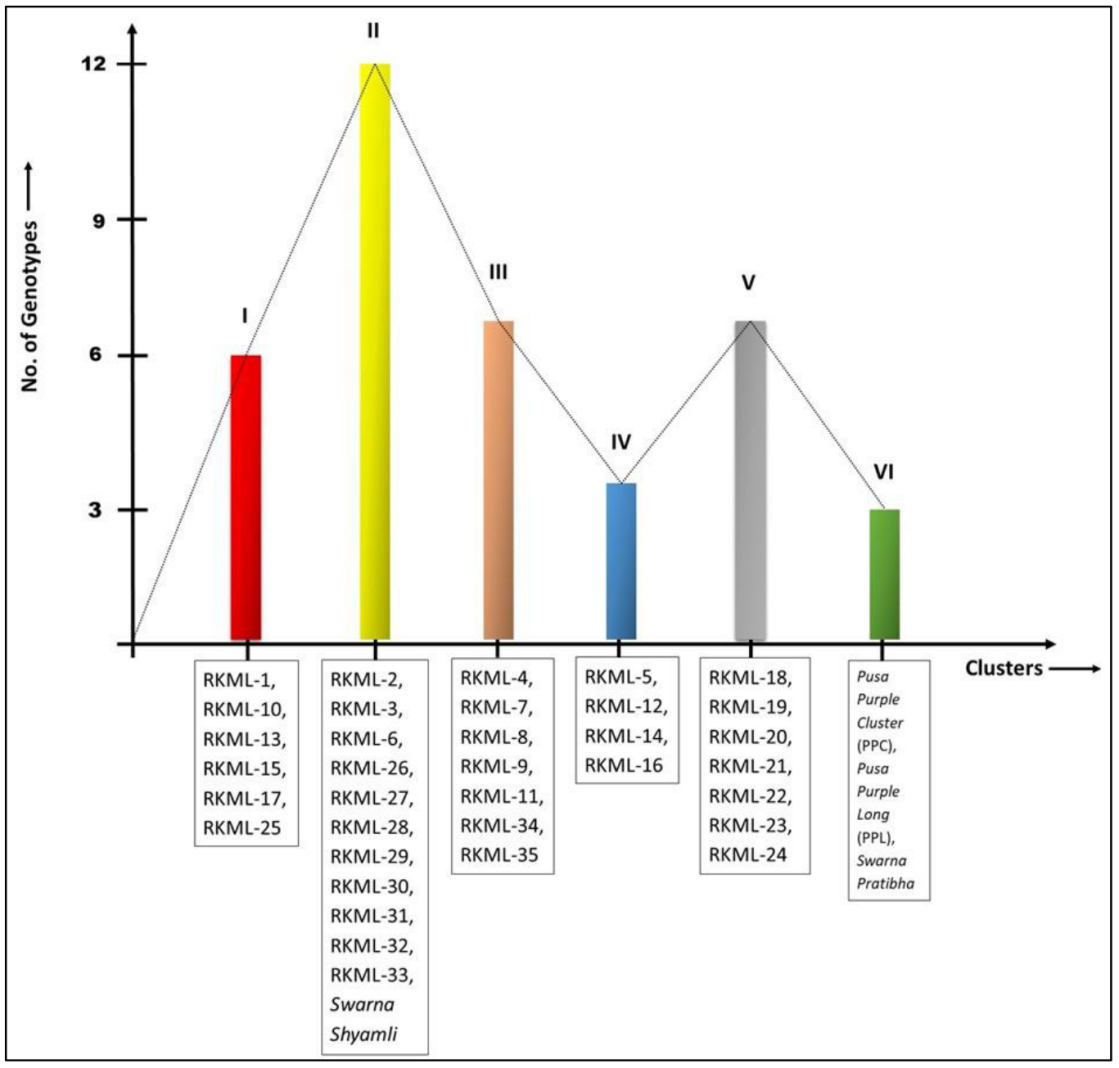
Grouping of 39 genotypes of brinjal into six different clusters on the basis of Euclidean Distance matrix.

Significant differences were observed across all investigated traits among the 39 indigenous brinjal lines through variance analysis, as presented in **Table 1**. Six distinct clusters were identified based on the *D* ^2^ statistic, indicating significant divergence among the lines (refer to **Figure 1**). The study revealed that the genetic divergence of germplasms did not exhibit a strong correlation with their geographical origins, as evidenced by the mean values that were distributed across the clusters, as presented in **Table 2**.

**Table 1:** Summary of Analysis of Variance for quantitative characters indicating considerable diversity for all 19 quantitative traits of 39 brinjal landraces.

Sl. No.	Characters	Sources of variation		
		Replication	Genotypes	Error
	DF	2	38	76
1	Plant Height (cm)	96.9	79.60**	26.61
2	Plant Spread North to South (cm)	368.36	96.84**	13.23
3	Plant Spread East to West (cm)	428.56	178.08**	37.8
4	Leaf Length (cm)	21.69	3.91**	1.97
5	Leaf Width (cm)	10.41	5.10**	0.9
6	No. of primary Branches	1.14	2.73**	0.49
7	Petiole Length (cm)	0.24	3.88**	0.42
8	Days to first flowering (DAT)	49.6	313.76	8.3
9	Days to 50 flowering (DAT)	91.34	308.15**	9.71
10	Fruit Initiation (DAT)	72.72	325.73**	23.43
11	Fruit Length (cm)	9.45	52.55**	2.99
12	Fruit Diameter (cm)	2	104.21**	8.46
13	Peduncle Length (cm)	4.23	4.23**	0.61
14	Fruits per plant (number)	5.79	262.74**	20.55
15	Fruit Weight (gram)	935.45	12006.53**	2008.02
16	Yield per plant (gram)	92781.37	408509.85**	101819.01
17	Total Yield (ton/ha)	57.53	129.22**	25.13
18	Seed per fruit (number)	65226.93	1431676.71**	24765.21
19	Plant Survival Rate (%)	824.43	560.86**	256.4

*,** significant at 5% and 1% levels, respectively; DF, degree of freedom.

**Table 2:** Cluster-wise mean values for 19 characters under study.

Clusters	DF	DF_50_	FD	FI	LL	LW	NPB	PH	PL	PSEW	PSNS	FL	FPP	FW	PDL	PH	PSR	SPF	TY	YPP
**Cluster I**	78.28	87.33	18.82	93.00	13.47	8.69	3.83	55.86	2.94	62.29	58.99	14.97	16.78	81.18	4.70	55.86	76.67	2506.39	14.71	1062.10
**Cluster II**	60.44	69.03	18.38	77.81	13.86	8.34	4.11	55.63	2.99	66.91	64.13	10.73	15.49	86.92	3.60	55.63	71.84	1894.36	16.07	935.83
**Cluster III**	81.38	86.62	28.00	94.52	16.19	11.44	5.62	63.91	5.02	75.15	70.50	17.12	8.04	188.80	5.94	63.91	88.57	2218.52	23.45	1450.14
**Cluster IV**	84.33	92.92	24.39	99.58	14.33	9.90	5.00	60.91	3.48	66.68	62.70	17.23	10.35	157.08	4.55	60.91	85.42	982.17	20.41	1378.50
**Cluster V**	78.38	86.81	14.35	95.95	14.66	9.25	4.00	58.17	3.33	75.10	69.42	9.22	30.38	43.02	3.84	58.17	87.04	1983.48	17.96	1013.33
**Cluster VI**	62.00	68.22	14.32	74.56	15.40	10.10	5.33	66.18	6.07	73.72	64.42	19.06	20.40	85.46	6.48	66.18	92.22	1600.78	23.00	1293.22

DF, days to flowering; DF50 days to 50% flowering; FI, fruit initiation; LL, leaf length (cm); LW, leaf width (cm); NPB, number of primary branches; Pl, petiole length (cm); FL, fruit length (cm); FD, fruit diameter (cm); FPP, number of fruit per plant; MFPP, number of marketable fruit per plant; FW, fruit weight (g); YPP, yield per plant (g); MYPP, marketable yield per plant (g); SPF, number of seed per fruit; TY, total yield (ton/ha).

The preeminent assemblage, Cluster II, was comprised of a total of 12 genotypes. Among these, four genotypes (namely, RKML-2, 3, 6, and 26) were chosen for the purpose of hybridization. The genotypes in Clusters III and V were both comprised of seven members. Four genotypes, namely RKML-7, 4, 11, and 34, were identified as potential candidates for further crossing from cluster III. Cluster I comprised six distinct genotypes, and the RKML-1 genotype was selected as the representative line for this cluster. Cluster IV comprised of four distinct genotypes, among which the RKML-5 line was chosen for the purpose of crossing. The present study reports the identification of three genotypes, namely Pusa Purple Cluster, Pusa Purple Long, and S. Pratibha, which were classified under Cluster VI. These genotypes were utilised as testers during the hybridization process. One of the testers from Cluster II, S. Shyamli, was chosen for the hybridization process. The selected lines and testers were sourced from separate clusters, indicating a significant divergence among the parental lines. This is a critical factor for future crossbreeding initiatives.

Distinct trait combinations were observed in each cluster. Cluster III, comprising of RKML-4, 7, 11, and 34, exhibited the most elevated cluster mean for nine distinct traits. On the other hand, Cluster IV, consisting of RKML-5, demonstrated the highest cluster mean for five distinct characteristics. In addition, the genotypes encompassed in this cluster exhibited the least amount of time required to attain 50% flowering and fruit initiation. The results of the study indicate that Cluster I exhibited the greatest seed count per fruit, whereas Cluster II (comprising of RKML-2, 3, 6, and 26) exhibited the shortest duration to flowering. Cluster V was observed to display the maximum count of fruits per plant, thereby indicating a superior level of productivity. The aforementioned observations provide evidence for the existence of significant genetic variation among the chosen parental strains, which is a crucial factor in achieving successful heterosis breeding.

### Genetic divergence based on SSR markers

A molecular characterization study was conducted on 14 genotypes to assess the genetic diversity among the parental lines. A total of twenty markers were employed in the study, revealing the presence of polymorphisms in eight of the 14 parental lines, thereby indicating the existence of genetic diversity (refer to **Supplementary Table 1**). The amplification profiles of DNA markers EM-131, EM-140, EM-155, EM-141, EM-117, EM-133, and EM-145 have been presented in **Supplementary Figures 2**–**8**. The markers utilised in this study revealed a total of nineteen distinct alleles, resulting in an average of 2.37 alleles per locus. The allelic frequencies were observed to range from 0.66 to 0.93, with an average of 0.83. Within the set of primers tested, EM-133 was found to possess the highest Polymorphism Information Content (PIC) value of 0.32, with EM-145 following closely behind with a PIC value of 0.27. The present study reveals a mean polymorphism of 56.25% within the parental population.

The population’s genetic diversity was observed to vary between 0.14 and 0.41, with an average value of 0.24, as presented in **Table 3**. The locus EM-133 exhibited the highest gene diversity (0.41), whereas the locus EM 120 displayed the lowest gene diversity (0.14). The application of the Unweighted Pair Group Method of Arithmetic Means (UPGMA) to the dataset resulted in the identification of five distinct clusters comprising the 14 genotypes under investigation, as presented in **Table 3** and **Supplementary Figure 1**.

**Table 3:** Distribution of Lines and Testers of Brinjal into five different clusters on the basis of UPGMA cluster analysis.

S. No.	Clusters	No. of Genotypes	Clustering of genotype on the basis of SSR markers/UPGMA
1.	I	1	RKML- 1
2.	II	1	RKML-2
3.	III	2	RKML-3 and RKML-4
4.	IV	4	RKML-5, RKML-6, RKML-7 and RKML-26
5.	V	6	Pusa Purple Cluster (PPC), Pusa Purple Long (PPL), SwarnaPratibha and RKML-34

The present study reports on the classification of six cultivars into Cluster V, which is the largest cluster. The cultivars included in this cluster are Pusa Purple Cluster, Pusa Purple Long, S. Shyamli, S. Pratibha, RKML-11, and 34. The fourth cluster comprised of specimens RKML-5, RKML-6, RKML-7, and RKML-26. Cluster III was established through the amalgamation of two distinct genotypes, namely RKML-3 and RKML-4. Conversely, Clusters I and II were found to be monotypic, consisting solely of RKML-1 and RKML-2, respectively. It is noteworthy that genotypes that pertain to a common cluster exhibit a higher degree of genetic proximity in comparison to those that are classified into distinct clusters. The comprehension of this concept is crucial in the identification of appropriate parental lines for the implementation of crossbreeding initiatives, with the objective of augmenting favourable characteristics in brinjal varieties.

### Magnitude of heterosis

**Figure 2:**
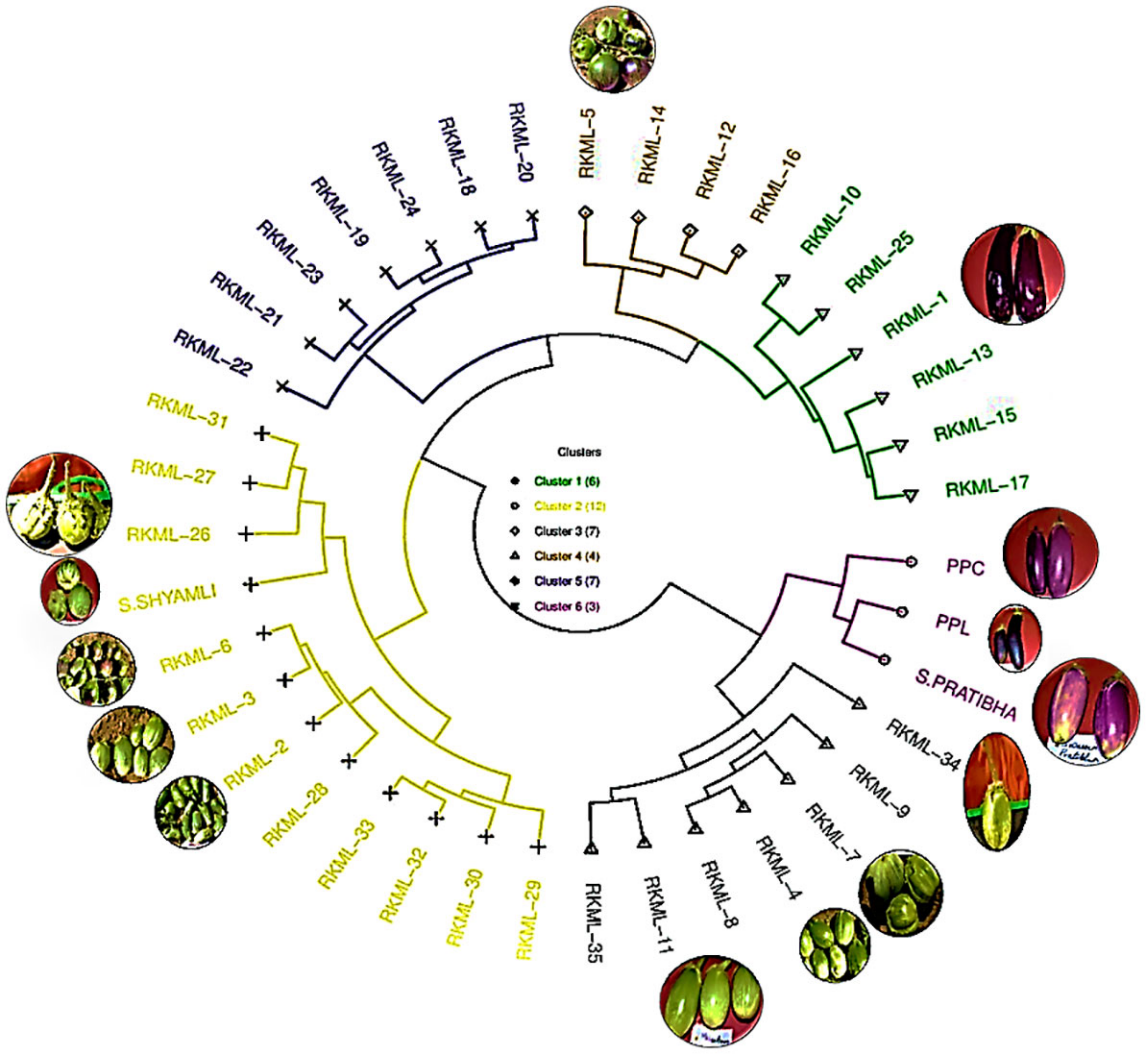
Selection of parents for Line X Tester mating design from six different clusters constructed on the basis of Euclidean Distance matrix for the present study.

In this study, we analysed the yield and related characteristics of fourteen parental lines, as depicted in **Figure 2**, along with forty hybrids. In our study, the primary objective was to elucidate the magnitude of heterosis for a variety of traits. Upon examination, we observed that heterosis was significant across all traits, as evidenced in **Table 4**. To ensure full understanding of our results, it is important to clarify the terms ‘mid-parent’, ‘superior parent’, and ‘standard check’:

**Table 4:** Estimates of percent Heterosis based on mid parent, better parent and standard parent of 40 Brinjal hybrids of yield characters.

Hybrids	YPP			MYPP			TY		
	Mid. Het.	Bet. Het.	Std. Het.	Mid. Het.	Bet. Het.	Std. Het.	Mid. Het.	Bet. Het.	STD. Het.
L1XT1	45.32**	25.70*	2.14	46.45**	15.32	-18.55	98.25**	97.54**	42.15*
L1XT2	44.50**	42.40*	-13.01	58.92**	39.91*	-25.32*	73.17**	62.71*	16.25
L1XT3	22.84	7.04	-14.52	38.51*	14.92	-29.23**	60.53*	57.77*	16.74
L1XT4	47.18**	17.24	17.24	50.90**	6.09	6.09	50.46*	28.98	28.98
L2XT1	14.07	-1.62	-20.05	9.44	-5.43	-33.21**	60.02*	53.32	10.34
L2XT2	-4.76	-6.46	-42.86**	-4.77	-6.50	-50.09**	25.83	22.85	-18.99
L2XT3	-32.15*	-41.04**	-52.92**	-24.26	-30.50	-57.20**	-7.55	-12.58	-35.32
L2XT4	43.57**	14.08	14.08	38.12**	4.58	4.58	88.87**	56.70**	56.70**
L3XT1	-33.56**	-33.66**	-45.93**	-32.72**	-34.42*	-51.22**	2.74	-2.41	-21.95
L3XT2	-45.19**	-52.05**	-60.92**	-41.77**	-49.99**	-62.80**	-24.08	-32.22	-45.79*
L3XT3	-33.17**	-33.85**	-46.08**	-27.16*	-33.43*	-50.48**	2.33	-1.50	-21.22
L3XT4	-28.61**	-35.21**	-35.21**	-27.17**	-36.50**	-36.50**	3.06	-7.25	-7.25
L4XT1	-12.78	-27.95**	-10.23	-8.88	-22.96*	-21.25*	34.17	8.59	26.31
L4XT2	-20.66*	-40.88**	-26.33*	-21.09	-39.94**	-38.61**	15.36	-11.17	3.33
L4XT3	-34.23**	-46.04**	-32.77**	-30.85**	-44.59**	-43.36**	-1.48	-19.40	-6.25
L4XT4	-0.42	-10.25	11.83	-0.02	-1.10	1.09	48.21**	37.81*	60.30**
L5XT1	-7.47	-21.83*	-7.89	-11.56	-26.14**	-22.17*	56.18**	34.39	34.13
L5XT2	12.36	-14.69	0.52	14.87	-13.47	-8.82	76.63**	43.90*	43.62*
L5XT3	11.76	-6.25	10.46	18.01	-6.52	-1.49	82.53**	58.93**	58.62**
L5XT4	-51.15**	-54.84**	-46.79**	-52.08**	-53.30**	-50.79**	-25.16	-25.23	-25.23
L6XT1	-31.91**	-33.31**	-43.48**	-30.40**	-35.87**	-46.26**	2.20	-7.79	-17.52
L6XT2	-38.56**	-47.13**	-55.19**	-39.11**	-50.17**	-58.24**	-25.54	-36.62	-43.31*
L6XT3	-25.71*	-27.86*	-38.86**	-24.37	-34.40**	-45.02**	5.80	-3.34	-13.53
L6XT4	-6.93	-14.03	-14.03	-5.52	-13.17	-13.17	22.91	16.42	16.42
L7XT1	-1.88	-9.74	-12.66	-1.26	-8.09	-24.67*	52.02*	37.39	22.44
L7XT2	12.20	-8.49	-11.44	11.24	-8.16	-24.72*	62.96**	38.91	23.80
L7XT3	13.00	3.13	-0.21	3.63	-9.25	-25.62*	67.93**	53.69*	36.96
L7XT4	-5.70	-7.22	-7.22	-9.02	-17.23	-17.23	32.45	25.25	25.25
L11XT1	29.84**	3.53	41.46**	27.53**	0.44	23.33*	91.25**	45.88**	99.78**
L11XT2	-10.45	-35.20**	-11.46	-9.44	-35.04**	-20.24	25.38	-8.55	25.24
L11XT3	-14.28	-32.09**	-7.21	-7.91	-30.86**	-15.10	24.47	-4.14	31.28
L11XT4	-39.50**	-47.61**	-28.41**	-38.31**	-44.03**	-31.28**	-14.79	-26.29	0.95
L26 XT1	61.61**	29.18**	75.37**	62.15**	23.44**	66.86**	96.53**	48.63**	108.67**
L26XT2	-12.85	-36.82**	-14.22	-12.25	-38.80**	-17.27	22.00	-11.71	23.95
L26XT3	30.42**	3.57	40.60**	31.45**	-4.34	29.32**	72.72**	31.88*	85.15**
L26XT4	-13.18	-24.61**	2.35	-13.17	-24.47**	2.10	24.38	6.49	49.50*
L34XT1	35.26**	11.51	39.68**	32.02**	4.62	26.32*	88.40**	45.49**	92.29**
L34XT2	35.39**	0.71	26.15*	30.51**	-5.90	13.62	79.97**	32.75*	75.45**
L34XT3	25.26**	2.56	28.47**	29.29**	-2.38	17.87	69.94**	32.54*	75.18**
L34XT4	15.18	3.57	29.73**	16.80*	6.77	28.91**	53.25**	34.61*	77.90**
SE	180.24	208.13	208.13	170.76	197.18	197.18	6.35	7.33	7.33

*,** Significant at 5% and 1% levels, respectively; YPP, yield per plant (g); MYPP, marketable yield per plant (g); TY, total yield (ton/ha).

‘Mid-parent’ refers to the mean value of a particular trait derived from both parent plants. This value serves as an essential benchmark for evaluating whether the hybrid offspring displays any superior performance over its parents.

‘Superior parent’ is the term assigned to the parent plant that exhibits superior performance for the trait in focus. Comparison with the superior parent enables us to ascertain whether the hybrid offspring has the capacity to exceed the best parent’s trait performance.

‘Standard check’ is a term used to denote a benchmark or reference variety that is widely recognized and against which all other varieties are compared during trials. This comparison allows us to comprehend the performance of a hybrid in relation to a well-established variety. Here, we used HABR 21, a popular genotype of the Jharkhand region as standard check.

Notably, the trait of ‘fruit yield per plant’ emerged as a critical area of interest. We observed that the heterosis extent for this particular trait was remarkably significant, thereby underscoring its pivotal role in heterosis breeding programmes.

The present study reports significant positive heterosis over the mid-parent for yield per plant and marketable yield per plant in ten hybrids. The present study investigated the phenomenon of heterosis in the hybrid RKML-26 X PPC. The results revealed that this hybrid exhibited a significant positive heterosis over the mid-parent for the traits yield per plant and marketable yield per plant. The magnitude of heterosis for yield per plant and marketable yield per plant was found to be 61.61% and 62.15%, respectively, which is quite remarkable. Significant heterosis was detected in seven hybrids for yield per plant and five hybrids for marketable yield per plant, when compared to the standard check. The present study reports on the significant positive heterosis observed in the hybrid RKML-26 X PPC for yield per plant (75.37%) and marketable yield (66.86%) over the superior parent.

Considerable positive heterobeltiosis was observed in three hybrids with respect to yield per plant, and in two hybrids for marketable yield per plant. The hybridization of RKML-1 and PPL resulted in the manifestation of the highest heterotic value, which was recorded at 42.40% and 39.91%. The present study investigated the total yield of the hybrid RKML1 X PPC and observed a significant positive heterosis of 98.25% over the mid-parent and 97.54% over the superior parent. In this study, a total of twenty hybrids were analysed for their trait expression and exhibited a notable degree of heterosis over the mid-parent. Additionally, fifteen of the hybrids displayed a statistically significant increase in positive heterobeltiosis. The present study reports the significant standard heterosis of twelve hybrids, among which the hybrid RKML-26 X PPC exhibited the highest degree of 108.67%. The results of this study suggest that there is a significant level of exploitable heterosis in brinjal. This highlights the potential of utilising heterosis breeding techniques to enhance the yield and other related traits in brinjal cultivars.

### Relationship between genetic distance and heterosis

While previous studies suggest that hybrid performance isn’t solely dependent on genetic distance, the relationship between heterosis and genetic distance is valid up to a specific threshold of genetic divergence (Satya and Debnath, 2009; Singh and Gupta, 2019; Sreewongchai et al., 2021). Heterosis can be derived from crosses between different genotypes of both cross-pollinated and self-pollinated species (Poehlman and Sleper, 1995; Satya and Debnath, 2009).

In this study, as presented in **Table 5**, a similar relationship between genetic distance and heterosis has been observed. When RKML-1 was crossed with PPC, the resulting hybrids demonstrated the highest degree of mid-parent heterosis and heterobeltiosis, correlated with a considerable measure of genetic distance. Conversely, when RKML-26 was crossed with PPC, the resulting hybrids not only displayed remarkable standard heterosis and performance but also showed a substantial genetic distance between them.

**Table 5:** Relationship of Genetic distance and *per-se* performance with heterosis for fruit yield in F_1_ hybrids of brinjal.

S. No.	Parents/Hybrids	Genetic Distance (GD)	Ranking on the basis of GD	Total Yield (q/ha)	Mid Parent Heterosis (%)	Better Parent Heterosis (%)	Standard Heterosis (%)
1	L1XT1	8.78	High	51.29	98.25**	97.54**	42.15*
2	L1XT2	5.9	Moderate	41.95	73.17**	62.71*	16.25
3	L1XT3	5.8	Moderate	42.12	60.53*	57.77*	16.74
4	L1XT4	6.9	Moderate	46.54	50.46*	28.98	28.98
5	L2XT1	8.0	High	39.81	60.02*	53.32	10.34
6	L2XT2	5.56	Moderate	29.23	25.83	22.85	-18.99
7	L2XT3	5.92	Moderate	23.34	-7.55	-12.58	-35.32
8	L2XT4	4.95	Moderate	56.54	88.87**	56.70**	56.70**
9	L3XT1	8.0	High	28.16	2.74	-2.41	-21.95
10	L3XT2	5.67	Moderate	19.56	-24.08	-32.22	-45.79*
11	L3XT3	5.7	Moderate	28.43	2.33	-1.50	-21.22
12	L3XT4	5.2	Moderate	33.47	3.06	-7.25	-7.25
13	L4XT1	6.99	Moderate	45.58	34.17	8.59	26.31
14	L4XT2	6.06	Moderate	37.28	15.36	-11.17	3.33
15	L4XT3	4.84	Moderate	33.83	-1.48	-19.40	-6.25
16	L4XT4	7.56	High	57.84	48.21**	37.81*	60.30**
17	L5XT1	8.03	Moderate	48.4	56.18**	34.39	34.13
18	L5XT2	6.49	Moderate	51.82	76.63**	43.90*	43.62*
19	L5XT3	7.2	High	57.24	82.53**	58.93**	58.62**
20	L5XT4	7.28	High	26.98	-25.16	-25.23	-25.23
21	L6XT1	8.1	High	29.76	2.20	-7.79	-17.52
22	L6XT2	6.66	Moderate	20.46	-25.54	-36.62	-43.31*
23	L6XT3	7.01	High	31.2	5.80	-3.34	-13.53
24	L6XT4	5.49	Moderate	42.01	22.91	16.42	16.42
25	L7XT1	5.91	Moderate	44.18	52.02*	37.39	22.44
26	L7XT2	5.63	Moderate	44.67	62.96**	38.91	23.80
27	L7XT3	5.18	Moderate	49.42	67.93**	53.69*	36.96
28	L7XT4	7.43	High	45.19	32.45	25.25	25.25
29	L11XT1	6.97	Moderate	72.09	91.25**	45.88**	99.78**
30	L11XT2	6.78	Moderate	45.19	25.38	-8.55	25.24
31	L11XT3	5.32	Moderate	47.37	24.47	-4.14	31.28
32	L11XT4	8.04	High	36.43	-14.79	-26.29	0.95
33	L26 XT1	5.69	Moderate	75.29	96.53**	48.63**	108.67**
34	L26XT2	6.11	Moderate	44.73	22.00	-11.71	23.95
35	L26XT3	5.37	Moderate	66.81	72.72**	31.88*	85.15**
36	L26XT4	6.0	Moderate	53.94	24.38	6.49	49.50*
37	L34XT1	6.75	Moderate	69.38	88.40**	45.49**	92.29**
38	L34XT2	7	Moderate	63.31	79.97**	32.75*	75.45**
39	L34XT3	5.44	Moderate	63.21	69.94**	32.54*	75.18**
40	L34XT4	8.1	High	64.19	53.25**	34.61*	77.90**

Positive correlations (r=0.29, 0.036) were estimated between genetic distance and better parent and standard heterosis, considering only the significant cases. These results indicate that when striving for normal heterosis, it may be beneficial to select hybrids that produce superior hybrids.

When comparing the yield assessment of hybrids with the genetic distance grouping of parent plants, it was observed that hybrids derived from crosses between parents with the highest genetic distance exhibited a higher degree of heterosis than those resulting from crosses between parents with the least genetic distance. This relationship further reinforces the importance of considering genetic distance in the pursuit of maximizing heterosis and yield potential in brinjal cultivation.

## Discussion

The crop of aubergine, which is highly variable in India, presents a multitude of landraces and commercial cultivars that showcase diverse morphological traits, specifically in relation to fruit characteristics (Tiwari et al., 2016). The present level of diversity observed in the population under consideration is deemed to be conducive for the purpose of selecting suitable parents possessing the desired traits, which is considered to be a crucial factor for the successful implementation of breeding programmes (Debnath et al., 2022). The inclusion of indigenous genotypes in breeding programmes can potentially augment the genetic diversity and improve hybrid vigor, as reported by Boyaci et al. (2015) and Muñoz-Falcón et al. (2009a). **Table 1** displays the significant diversity observed in 39 brinjal landraces across 19 quantitative traits, as discovered in the present study. A cohort of ten lines, derived from separate clusters, was chosen based on their diversity and potential for use in future crossbreeding initiatives. The four testers were also selected for their distinct origins. This selection process was conducted with the aim of maximising the genetic variability of the resulting progeny.

The study conducted by Premabati et al. (2016) provides evidence that the crossing of genotypes from distant clusters results in a considerable level of heterotic response. Clusters III and IV displayed the most elevated cluster means for nine and five traits, respectively. This suggests that genotypes originating from these clusters would demonstrate substantial heterosis and genetic diversity for economically significant brinjal traits, as reported by Hazra et al. (2010); Madhavi et al. (2015), and Tiwari et al. (2016).

The molecular characterization of high-quality germplasm is an essential aspect of formulating breeding tactics and recognising exceptional complementary lineages. The utilisation of molecular markers, such as Simple Sequence Repeats (SSRs), for the purpose of genetic diversity analysis has been deemed a cost-effective approach. This is due to the high variability, enhanced genome coverage, repeatability, automation potential, neutrality, and immunity to environmental disturbances that SSRs offer (Prohens et al., 2008; Muñoz-Falcón et al., 2009b; Adeniji et al., 2012; Hurtado et al., 2012; Bhandari et al., 2017). The present study reports lower average PIC value and genetic diversity compared to earlier investigations, which may be attributed to variations in sample size, materials, and marker count as previously suggested (Hurtado et al., 2012; Ge et al., 2013; Chourey et al., 2017).

The phenomenon of heterosis is characterised by the enhanced performance of hybrid progeny in multiple domains, including but not limited to yield, adaptability, resistance to pests and diseases, overall robustness, and quality. The present investigation evaluated the performance of hybrid RKML1 X PPC. Results showed that this hybrid manifested the most noteworthy positive effect over the mid-parent and better parent. Approximately 50% of the crosses displayed considerable heterosis over the mid-parent, while 37% exhibited significant heterobeltosis in a positive direction. These findings were reported by Patel et al. (2013) and Mistry et al. (2018). The findings of Singh et al. (2023) indicate that the presence of favourable heterosis in hybrids may offer opportunities for the utilisation of genes that can facilitate the development of cultivars with superior yield potential.

The comprehension of the correlation between genetic diversity and heterosis is of great significance for the advancement of inbred lines and plant breeding, with the aim of enhancing the efficacy of germplasm utilisation (Mindaye et al., 2015; Chand et al., 2022). Several investigations have documented conflicting outcomes regarding the correlation between genetic divergence and heterosis in instances where the genetic divergence is substantial (Pandey et al., 2015; Tomkowiak et al., 2020). The investigation conducted by Sreewongchai et al. (2021), Silva et al. (2020), and Hazra et al. (2010) has confirmed that the hybrids produced from the crosses between RKML-1 and PPC exhibit the greatest levels of mid-parent heterosis, heterobeltiosis, and a high genetic distance for fruit production. Conversely, the hybrids generated from RKML 26 and PPC display the highest standard heterosis and per se performance for fruit yield, despite their modest genetic distance. The present study elucidates the possibility of enhancing heterosis in brinjal breeding by employing divergent lines with moderate genetic divergence.

## Conclusion

Our understanding of heterosis and its underlying mechanisms remains limited, with the exploitation of F_1_ hybrid vigor being our primary approach for generating heterosis. Therefore, Simple Sequence Repeats (SSRs) present an advantageous method for identifying parental components contributing to heterotic effects due to their high polymorphism, which allows differentiation even among closely related individuals. The study provided compelling evidence that economically superior hybrids are typically a product of more genetically distinct parents. However, in the case of brinjal, a remarkable observation was that hybrids derived from crossings between parents with moderate genetic distance exhibited more heterosis than those resulting from parents with a very high genetic distance. These findings suggest that genetic distance can serve as a reliable predictor of heterosis in parents, but only up to a certain threshold. As we look towards the future, a key area of focus should be a deeper exploration of the relationship between genetic distance and heterosis. Additionally, understanding the precise genetic mechanisms and factors driving heterosis could lead to more predictive and strategic hybrid breeding programs. Furthermore, studying the interplay of genetic diversity with environmental factors may offer insights into how to increase crop yield stability under variable conditions. Lastly, advancing molecular marker technology, such as SSRs, and integrating it with advanced genomic and bioinformatic tools could provide a more nuanced understanding of the heterotic effect, eventually leading to more effective and efficient strategies for crop improvement.

## Data availability statement

The original contributions presented in the study are included in the article/**Supplementary Material**. Further inquiries can be directed to the corresponding author.

## Author contributions

SD and NR: conceptualization, data curation, investigation, supervision, and manuscript writing and editing. KP, FK, BP, AS, MK VS, VK, PJP and ML: data analysis, investigation, manuscript writing and editing. All authors contributed to the article and approved the submitted version.
