# Supplementary material for: Optimizing hybrid vigor: a comprehensive analysis of genetic distance and heterosis in eggplant landraces
Source: Front Plant Sci. 2023 Aug 31;14:1238870. doi: 10.3389/fpls.2023.1238870 (PMC10501132; doi:10.3389/fpls.2023.1238870)
Supplement: Supplementary file 2 [file Image_1.pdf]

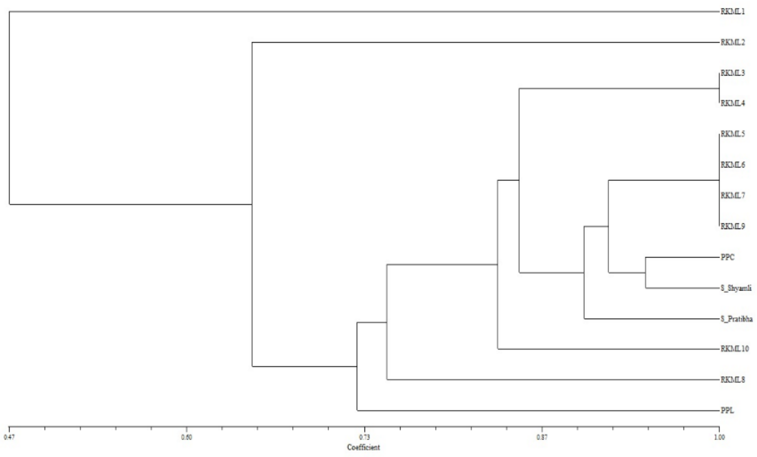

Supplementary fig.1: Dendrogram constructed from the SSR data, using Jaccard's coefficient of similarity and UPGMA clustering

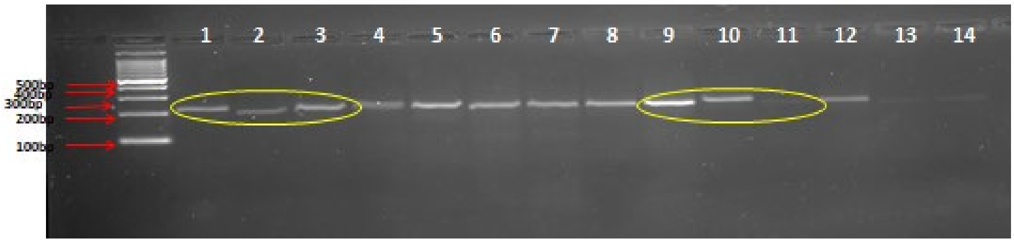

Supplementary fig. 2: Banding pattern of SSR locus EM-131 for14 parental lines of brinjal

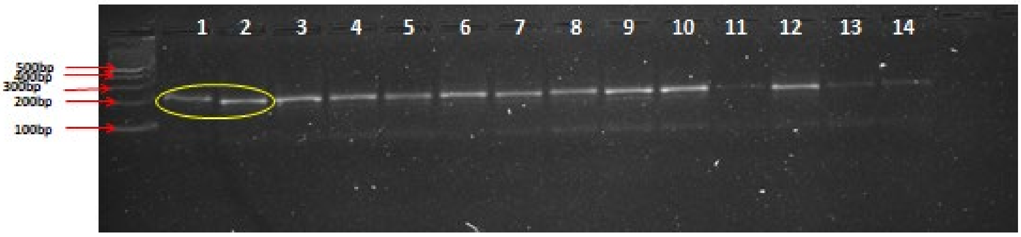

Supplementary fig. 3: Banding pattern of SSR locus EM-140 for14 parental lines of brinjal

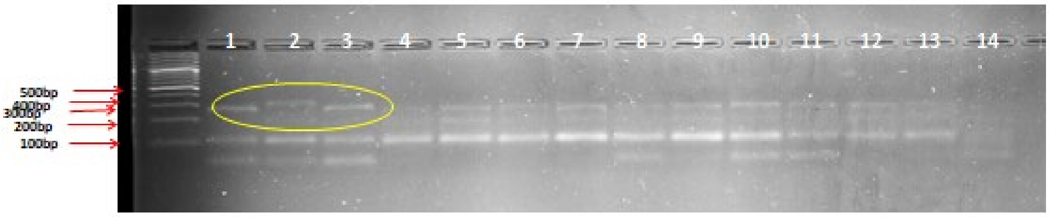

Supplementary fig. 4: Banding pattern of SSR locus EM-155 for14 parental lines of brinjal

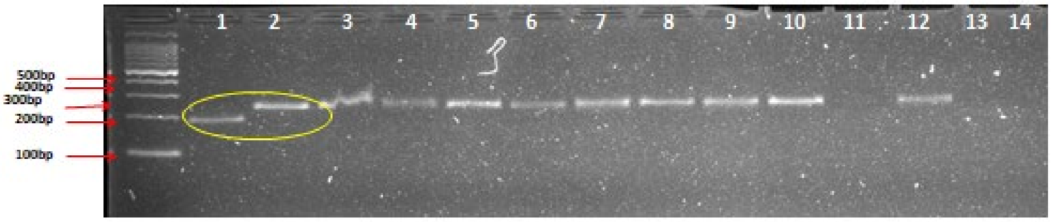

Supplementary fig. 5: Banding pattern of SSR locus EM-141 for14 parental lines of brinjal

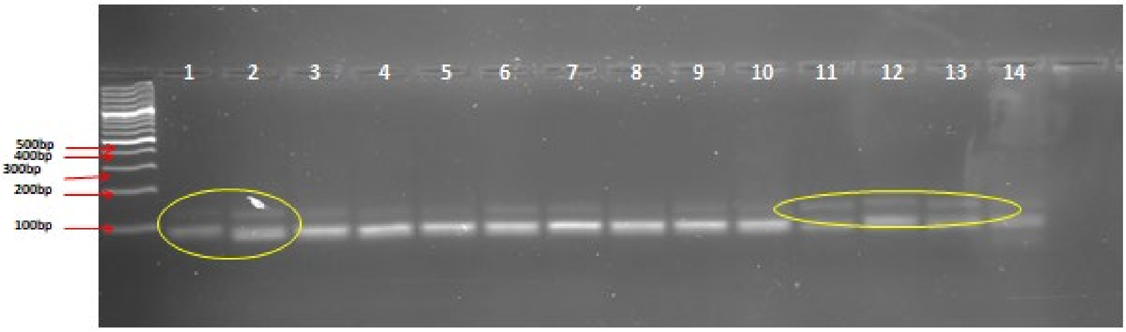

Supplementary fig. 6: Banding pattern of SSR locus EM-117 for14 parental lines of brinjal

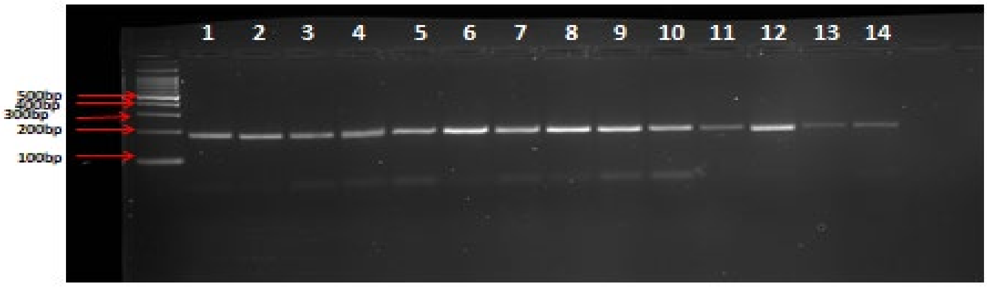

Supplementary fig. 7: Banding pattern of SSR locus EM-133 for14 parental lines of brinjal

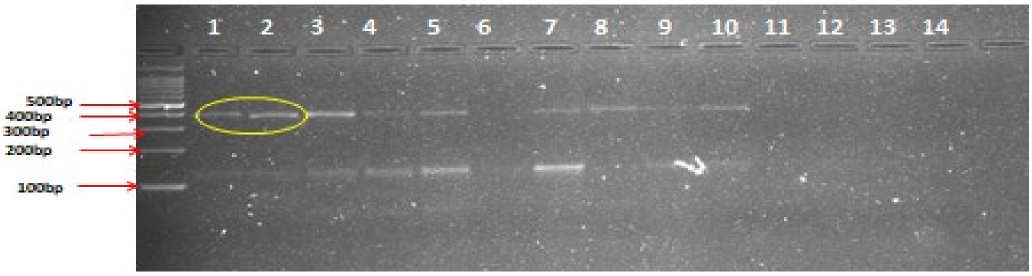

Supplementary fig. 8: Banding pattern of SSR locus EM-145 for14 parental lines of brinjal

Left most lane corresponds to DNA ladder and amplified DNA samples were loaded in the lanes following the order of genotypes as

1- RKML1	8- RKML 11
2- RKML 2	9- RKML 26
3- RKML 3	10 - RKML 34
4- RKML 4	11- PPC
5- RKML 5	12- PPL
6- RKML 6	13- S. Pratibha
7- RKML 7	14- S. Shyamli
