# Supplementary material for: Optimizing hybrid vigor: a comprehensive analysis of genetic distance and heterosis in eggplant landraces
Source: Front Plant Sci. 2023 Aug 31;14:1238870. doi: 10.3389/fpls.2023.1238870 (PMC10501132; doi:10.3389/fpls.2023.1238870)
Supplement: Supplementary file 3 [file Table_1.docx]

**Supplementary Table. 1:** Sequences of Forward and reverse primers of SSR markers used for the molecular characterization of fourteen parental lines of brinjal

| **S. No.** | **Primer Name** | **Primer Sequence 5'-3'** | **Anneal. Temp. (^0^C)** | **Product size ( in bp)** | **Amplified Product (in bp)** | **Alle-les** | **% Poly-morphism** | **Major Allele Frequency** | **Gene Diversity** | **PIC value** |
| --- | --- | --- | --- | --- | --- | --- | --- | --- | --- | --- |
| **1** | **EM 117** | GATCATCACTGGTTTGGGCTACAA AGGGGAGAGGAAACTTGATTGGAC | 65 | 123 | 115-130 | 3 | 66.67 | 0.93 | 0.25 | 0.24 |
| **2** | **EM 131** | TCTGGGACACCAAGTGAAAAATCA CATGCGTGAGTTTTGGAGAGAGAG | 65 | 213 | 200-230 | 3 | 66.67 | 0.86 | 0.25 | 0.24 |
| **3** | **EM 133** | GCGGATCACCTGCAGTTACATTAC TCCTTTGACCTATAGTGGCACGTAGT | 65 | 177 | 190-195 | 2 | 50 | 0.71 | 0.41 | 0.32 |
| **4** | **EM 140** | CCAAAACAATTTCCAGTGACTGTGC GACCAGAATGCCCCTCAAATTAAA | 65 | 268 | 200-220 | 3 | 66.67 | 0.86 | 0.25 | 0.24 |
| **5** | **EM 141** | TCTGCATCGAATGTCTACACCAAA AAAAGCGCTTGCACTACACTGAAT | 65 | 228 | 195-250 | 2 | 50 | 0.86 | 0.14 | 0.13 |
| **6** | **EM 155** | CAAAAGATAAAAAGCTGCCGGATG CATGCGTGAGTTTTGGAGAGAGAG | 65 | 248 | 280-300 | 2 | 50 | 0.86 | 0.14 | 0.13 |
| **7** | **EM 145** | CAGTGCTACATAAATTGAGACAAGAGG GGAGGTACAACGGATTTTCATATGGT | 65 | 369 | 395-400 | 2 | 50 | 0.66 | 0.32 | 0.27 |
| **8** | **EM 120** | GGATCAACTGAAGAGCTGGTGGTT CAGAGCTTCAATGTTCCATTTCACA | 65 | 160 | 165-170 | 2 | 50 | 0.86 | 0.14 | 0.13 |
